# A plastic surgery approach to Meige syndrome: Botulinum toxin for rebalancing facial dystonia and aesthetics – A case report

**DOI:** 10.1016/j.jpra.2026.03.010

**Published:** 2026-03-11

**Authors:** Xiao Long

**Affiliations:** Department of Plastic Surgery, The Xiaolifeidao Medical Cosmetology Hospital, Guiyang, Guizhou 550081, China

**Keywords:** Meige syndrome, Botulinum toxin, Facial dystonia, Aesthetic surgery, Muscle rebalancing

## Abstract

Meige syndrome is a rare segmental dystonia characterized by blepharospasm and oromandibular dystonia, often leading to significant functional and aesthetic impairment. This report details a 59-year-old female patient with a 16-year history whose symptoms were refractory to prior drug therapies. An individualized botulinum toxin type A (BTX-A) injection protocol was devised based on detailed facial muscle mapping and plastic surgery principles of muscle balance. Following treatment, the patient showed marked improvement in facial spasms within 1 week, with resolution of nocturnal headaches and notable correction of aesthetic concerns arising from dystonia, including facial asymmetry and the involuntary “gummy smile”. The gingival exposure during dystonic contraction was reduced from >4 mm (classified as severe gingival smile) to 1 mm. Social and eating functions were restored. This case suggests that a BTX-A treatment strategy guided by plastic surgery principles, which addresses both functional symptoms and aesthetic disharmony, may provide an effective and safe office-based treatment option for Meige syndrome. Further studies with larger cohorts are warranted to validate this protocol.

## Introduction

Meige Syndrome (MS) is a segmental dystonia causing involuntary spasms of periorbital and perioral muscles.^1^ Beyond functional disability, it creates aesthetic disturbances—deep wrinkles, asymmetry, abnormal smiles—that align with conditions managed in aesthetic plastic surgery clinics. The involuntary “gummy smile” or gingival smile (GS) commonly seen in oromandibular dystonia, characterized by excessive gingival exposure, is a typical example.^2^ However, plastic surgeons' experience with MS is limited. Botulinum toxin type A (BTX-A) injection is a well-established, first-line treatment for focal and segmental dystonias, including MS.^3^ This case report illustrates how applying aesthetic injection principles can achieve dual functional and cosmetic improvement in this neurological disorder.

## Case presentation

A 59-year-old female presented to our clinic in June 2023. She had a 16-year history of worsening involuntary eye squeezing, frowning, and perioral twitching, exacerbated by speech and eating, accompanied by nocturnal headaches. Previous neurological treatments provided incomplete relief.

Physical examination at the time of treatment revealed the following findings:

## Static assessment (at rest)

Symmetric forehead lines with moderate wrinkles were present. The glabellar and medial canthal wrinkles were mild to moderate, while severe transverse wrinkles were noted at the nasal root, alongside lateral canthal fish-tail wrinkles. Mild vertical lip lines were observed in the upper and lower lip vermilion borders.

## Dynamic assessment (with movement)

### Upon eye closure

Frequent twitching of the bilateral eyelids and eyebrows, accompanied by continuous nose wrinkling, was observed. This action deepened the wrinkles at the medial and lateral canthi, as well as the transverse nasal root wrinkles. Severe oblique wrinkles appeared in the middle of the nasal dorsum. Concurrently, her lips frequently contracted involuntarily and pursed, which further deepened the vertical lip lines (Video 1, segment: pre-treatment, eyes closed).

### Upon eye opening and speech

**Figure 1:**
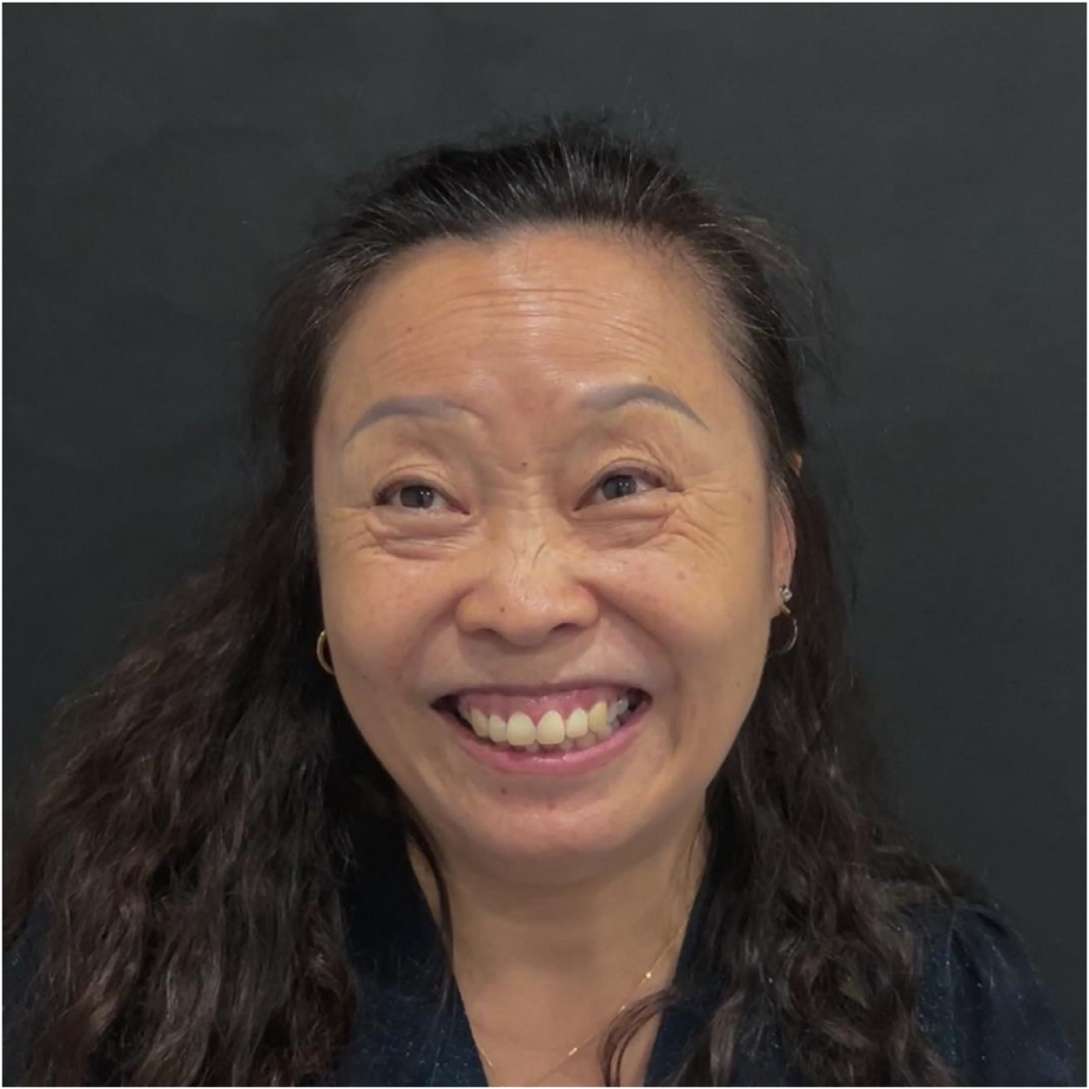
Pre-treatment: state when speaking with eyes open during the first medical visit, showing severe involuntary gingival exposure and leftward oral commissure deviation.

Involuntary contractions of the frontalis and glabellar muscles caused the eyebrows to rise and deepened the forehead lines. The upper eyelids were passively elevated, leading to widened palpebral fissures, overexposure of the eyeballs, and a resultant “staring” appearance. This was accompanied by dilated nares, involuntary lip twitching, and a forced, dystonic smile-like posture with excessive elevation of the upper lip (Video 1, segment: pre-treatment, speaking). The latter resulted in complete exposure of the gums, forming a severe, involuntary gingival display. Gingival exposure was measured in millimeters from the inferior border of the upper lip to the gingival margin on standardized frontal view photographs taken during maximal involuntary dystonic smile, and exceeded 4 mm, corresponding to a severe gingival smile (severe GS) according to established classification systems.^2^ The nasolabial folds were deepened, particularly on the left side, and a slight leftward deviation of the mouth corner was observed (Figure 1).

## Treatment

### Treatment protocol

Botulinum toxin type A (BTX-A, Prosigne®, Lanzhou Institute of Biological Products, China) was used. The dosing was based on the manufacturer's recommendations and clinical experience with this formulation. A 100 U vial was reconstituted with 2.0 mL of 0.9% normal saline. Using a 34 G needle, injections were administered subcutaneously/intramuscularly.

### Rationale for injection protocol design

**Figure 2:**
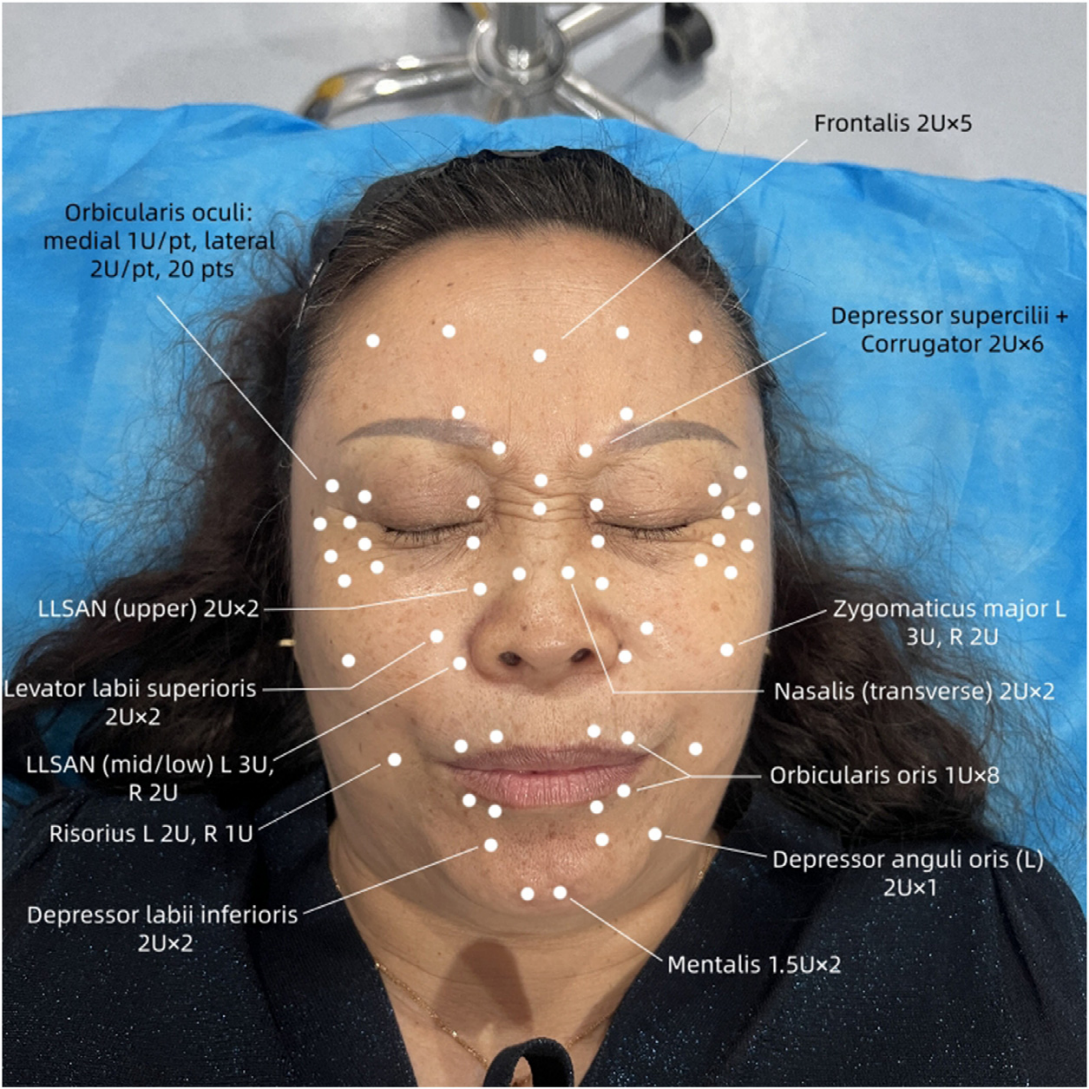
Frontal photograph of the patient with marked injection points according to the protocol in Table 1.

The protocol was individualized based on the dynamic assessment (Video 1). Key findings guiding site selection and dosing included: 1) Prominent hyperactivity of the levator labii superioris and levator labii superioris alaeque nasi during dystonic smile, targeted as primary sites to reduce severe gingival exposure; 2) Asymmetric perioral contraction with left-sided predominance, leading to oral commissure deviation, addressed by a higher dose to the left depressor anguli oris (as detailed in Table 1) to re-establish horizontal balance, exemplifying the principle of asymmetric dosing; 3) Involvement of the risorius (which also received asymmetric dosing, see Table 1) and depressor labii inferioris in the observed perioral twitching and lip pursing, targeted to achieve broader perioral muscle equilibrium. The complete injection protocol, including all asymmetric dosing strategies, is presented in Table 1. The approximate locations of the injection points and corresponding doses are illustrated in Figure 2.

**Table 1:** Individualized injection protocol of botulinum toxin type A (BTX-A, Prosigne®) for Meige syndrome.

Injection site	Muscle action/Rationale	Injection level	No. of points	Dose per point (U)	Total dose (U)
Frontalis	Forehead wrinkling; involuntary brow movement	Intramuscular	5	2.0	10
Depressor supercilii and Corrugator supercilii	Glabellar vertical lines; frowning action	Intramuscular	6	2.0	12
Orbicularis oculi (medial and lateral canthus)	Blepharospasm and eyelid twitching	Subcutaneous	20	Medial:1.0, Lateral: 2.0	36
Nasalis (transverse part)	Wrinkling at nasal root and dorsum	Subcutaneous	2	2.0	4
Levator labii superioris alaeque nasi (upper segment)	Nostril dilation	Intramuscular	2	2.0	4
Orbicularis oris (upper and lower lips)	Lip twitching and pursing	Subcutaneous	8	1.0	8
Mentalis	Involvement in lower lip movement	Intramuscular	2	1.5	3
Levator labii superioris alaeque nasi (middle and lower segment)	Excessive upper lip elevation (“gummy smile”)	Subcutaneous	2	Left 3.0 Right 2.0	5
Levator labii superioris	Excessive upper lip elevation (“gummy smile”)	Subcutaneous	2	2.0	4
Zygomaticus major	Excessive lip corner elevation (dystonic grin)	Subcutaneous	2	Left 3.0 Right 2.0	5
Risorius	Lateral retraction of oral commissure	Subcutaneous	2	Left 2.0 Right 1.0	3
Depressor labii inferioris	Excessive lower lip depression (dystonic grin)	Subcutaneous	2	2.0	4
Depressor anguli oris (left)	Hyperactivity relative to right side, causing leftward oral commissure deviation during dystonia	Subcutaneous	1 (left only)	2	2
**Total**			**54 points**		**100 U**

**Note:***U* = units of botulinum toxin type A. The injection sites and doses were individualized based on the assessment of static and dynamic facial muscle hyperactivity, aiming to achieve muscular rebalancing.

## Results

The patient's clinical course was documented with serial video and photographic assessments. Validated dystonia rating scales were not employed in this clinic-based aesthetic setting; assessment relied on clinical observation, patient report, and photographic measurement of gingival exposure.

### One week post-treatment

**Figure 3:**
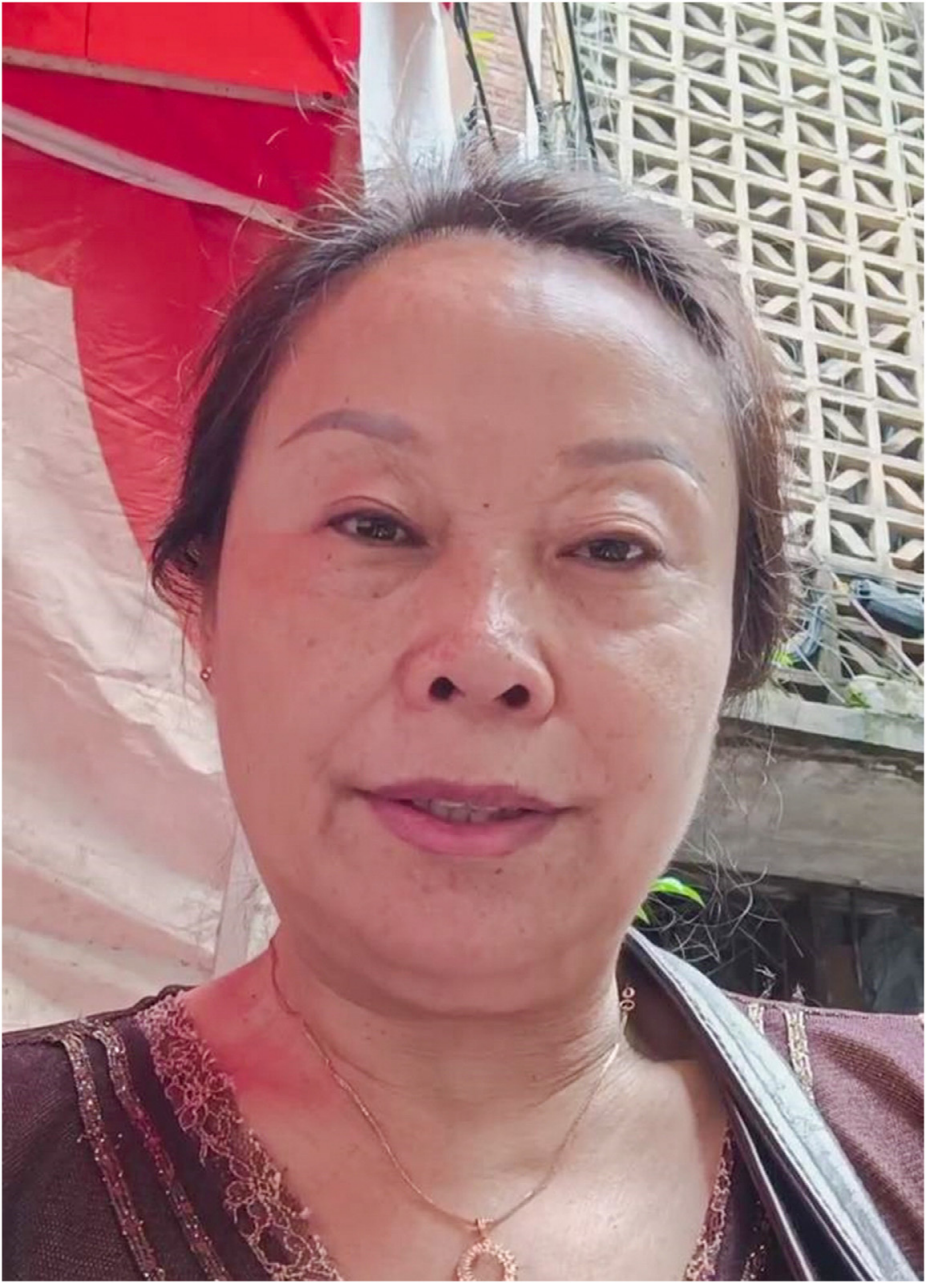
Seven days after the first botulinum toxin injection, showing marked improvement in facial spasms and gingival exposure.

Symptom relief was notable within 1 week, with a dramatic reduction in blepharospasm and perioral hyperkinesis (Figure 3 and Video 1, segment: 7 days post-treatment).

### One month follow-up

The therapeutic effect was fully established. Nocturnal headaches resolved. Gingival exposure was measured as the vertical distance from the inferior border of the upper lip to the gingival margin on standardized frontal view photographs, captured during maximal involuntary dystonic smile. This measurement was reduced from >4 mm (severe GS) pre-treatment to 1 mm post-treatment. She was specifically instructed to report any symptoms of eyelid ptosis, diplopia, dysphagia, or facial weakness, and none were reported during scheduled follow-ups.

### Four-month follow-up

Clinical and aesthetic improvement remained stable, as documented in video 1 (segment: 4 months post-treatment). The patient reported unimpeded social interaction and normal eating function.

### Seven-month follow-up & re-injection

Mild recurrence of symptoms was observed (Video 1, segment: 7 months post-treatment). Based on the sustained good response and duration of effect from the first treatment, and to explore the minimal effective dose while mitigating potential risks associated with cumulative exposure, a reduced total dose of 70 U BTX-A (Prosigne®) was administered for the second session. This subsequent treatment successfully alleviated the recurrent symptoms.

## Discussion

This case highlights the unique value plastic surgeons can add to MS management by integrating deep knowledge of facial anatomy, antagonistic muscle balance, and aesthetic outcome optimization.^4^ Our approach mirrors aesthetic practices, such as differential dosing for asymmetry correction, applying the same “muscle rebalancing” logic used in cosmetic procedures.^5^ The successful management of the concomitant severe gingival smile underscores this synergy, achieving an aesthetic target (<2–3 mm gingival exposure)^2^ alongside functional improvement.

It is important to note that the BTX-A formulation used (Prosigne®) is produced in China. Long-term studies have established the efficacy and safety of different BTX-A formulations (e.g., abobotulinumtoxinA and onabotulinumtoxinA) in treating blepharospasm and Meige syndrome.^6^ While direct comparative efficacy studies with Prosigne® are limited, differences in diffusion characteristics between formulations are known and may influence dosing and injection patterns.^7^ Our dosing was determined empirically based on this specific product and the patient's individual muscle mass and spasm severity.

Compared to systemic neurological treatments,^8^ this office-based, minimally invasive approach offers a targeted solution with rapid recovery, suitable for patients concerned with functional and aesthetic facial distress. The injection protocol, involving 54 points and 100 U of Prosigne® in this case, was complex. While no complications occurred, risks such as brow ptosis, lip weakness, or dysphagia are acknowledged, necessitating meticulous anatomical precision and possibly ultrasound guidance in complex cases.^9^

Limitations: This is a single-case report, which precludes generalization of results. The lack of validated dystonia or patient-reported outcome scales is a limitation. Future studies with larger cohorts, standardized scales, and longer follow-up are needed. The use of a specific BTX-A formulation (Prosigne®) may affect the reproducibility of dosing with other products.

## Conclusion

This case illustrates a potential office-based treatment framework and suggests that a botulinum toxin injection protocol, guided by plastic surgery principles of muscle rebalancing and aesthetic analysis, may effectively address both functional and cosmetic impairments in Meige syndrome. It highlights a promising interdisciplinary role for plastic surgery in managing complex facial movement disorders. The protocol's reproducibility and efficacy require validation through larger, controlled studies.

## Patient consent

Written informed consent was obtained from the patient for publication of this case report and any accompanying images/video.

## Ethical approval

Approval was obtained from the Independent Ethics Committee of Xiaolifeidao Medical Cosmetology Hospital (Approval No 2025-001). All procedures adhered to the Declaration of Helsinki.

## Funding source

This research did not receive any specific grant from funding agencies in the public, commercial, or not-for-profit sectors.

## Declaration of competing interest

None declared.

## Appendix Supplementary materials

Video 1. Dynamic clinical course of Meige syndrome in a 59-year-old female treated with botulinum toxin. Segment 1: Pre-treatment state with eyes closed, showing forceful blepharospasm and eyebrow twitching. Segment 2: Pre-treatment state when speaking with eyes open, demonstrating a left-dominant hyperkinetic dystonic contraction, manifesting as severe involuntary gingival exposure (“gummy smile”) and lip pursing. Segment 3: Seven days after the first botulinum toxin injection, showing marked reduction in spasms. Segment 4: Four months after the first injection, showing sustained improvement. Segment 5 & 6: Seven months after the first injection, showing mild recurrence of symptoms with eyes closed (5) and while speaking (6). Image, video 1 dummy alt text
